# Genomic characterisation of the metal tolerance protein gene family and elucidation of functional role in heavy metal tolerance and accumulation in *Coptis chinensis*

**DOI:** 10.3389/fpls.2025.1658134

**Published:** 2025-10-08

**Authors:** Yi Zhang, Lili Wang, Jinke Zhang, Chenxi Wang, Yue Fan, Haohao Cao, Huanyu Zhang, Yuchen Ma, Haoyu Wang, Jiaxin Kang, Yige Jiao, Xinxin Shangguan, Xiaoli Li, Kedong Xu

**Affiliations:** ^1^ Key Laboratory of Plant Genetics and Molecular Breeding, Zhoukou Normal University, Zhoukou, China; ^2^ Zhoukou Normal University Henan Key Laboratory of Crop Molecular Breeding and Bioreactor, Zhoukou, China; ^3^ Zhoukou Normal University Henan Plant Gene and Molecular Breeding Engineering Research Center, Zhoukou, China; ^4^ Zhoukou Normal University Henan Crop Molecular Design Breeding and Cultivation Engineering Technology Research Center, Zhoukou, China; ^5^ Zhoukou Academy of Agricultural Sciences, Zhoukou, China

**Keywords:** metal tolerance protein, *Coptis chinensis*, heavy metal tolerance, Cd, Δ*ycf1* yeast

## Abstract

Metal tolerance protein (MTP) family members, functioning as plant divalent cation transporters, play essential roles in maintaining heavy metal homeostasis and tolerance. This study presents a novel comprehensive genomic characterisation of the *MTP* gene family in *Coptis chinensis*, involving systematic identification and functional annotation. A total of 25 *CcMTP* genes were identified and classified into three major subfamilies based on phylogenetic analysis. Conserved motif profiling and gene structure annotation revealed both conserved and divergent features among the subfamilies. Genomic collinearity analysis identified one tandemly duplicated gene pair (*CcMTP12*/*CcMTP20*) in *C. chinensis*. The promoter regions of *CcMTP* genes were enriched with cis-acting elements associated with phytohormones, light, and growth and development. Gene expression analysis showed that several *CcMTPs* were significantly upregulated in response to cadmium stress across different tissues. *CcMTP11, CcMTP16*, and *CcMTP24*, which exhibited high expression in roots, stems, and leaves, conferred enhanced tolerance to multiple heavy metals. Notably, *CcMTP24* promoted Δ*ycf1* yeast growth under Cd, iron, zinc, and manganese stress and reduced Cd accumulation in yeast. Collectively, pivotal *CcMTPs*, such as *CcMTP24* identified in this study, may provide mechanistic insights to elucidate the roles of MTPs in heavy metal tolerance in *C. chinensis*.

## Introduction

Heavy metal pollution poses a great threat to plant growth and development, human health, and environmental safety (Bari et al., 2021; Fan et al., 2023). Some essential heavy metals, such as iron (Fe), zinc (Zn), and manganese (Mn), play vital roles at low concentrations in plant metabolism and development (Fan et al., 2023). However, excessive levels of these micronutrients result in toxic effects on plants and can even impact human health (Lefèvre et al., 2018). In contrast, other heavy metals, such as arsenic (As), lead (Pb), cobalt (Co), and cadmium (Cd) are considered non-essential elements that can cause toxicity in crop plants even at very low concentrations and pose a severe health threat to humans through the food chain (Dai and Zhou, 2025). Among all heavy metals, Cd ranks as the most prevalent and hazardous inorganic pollutant in arable soil (Sun et al., 2014). In plants, Cd exposure results in easily recognisable symptoms, such as chlorosis and stunted growth. Cd contamination also causes a decline in leaf transpiration, inhibition of nutrient element absorption and distribution, accumulation of reactive oxygen species (ROS), and disruption of stomatal movements, leading to physiological and morphological damage in plants. Moreover, an excessive amount of Cd in plants typically hinders growth and may result in death (Lal et al., 2019; Zhao et al., 2022).

To overcome Cd toxicity, plants have evolved complex and sophisticated regulatory mechanisms (Shahid et al., 2017; Yokosho et al., 2021). Among these mechanisms, two crucial strategies for reducing or eliminating the accumulation and toxicity of Cd in plant cells are hindering the inward flow of Cd and enhancing its outward flow (Han et al., 2023; Kanwal et al., 2024). Numerous heavy metal transporter families are responsible for the absorption, accumulation, and transportation of Cd in plants (Yang et al., 2022; Bari et al., 2021). The cation diffusion facilitator (CDF) family, members of which act as integral membrane transporters for divalent cations (Mn^2+^, Zn^2+^, Fe^2+^, Co^2+^, Cd^2+^, and Ni^2+^), is involved in the efflux of these ions, either transporting them from the cytoplasm into vacuoles or to the extracellular space (Gustin et al., 2011). Due to their evolutionary conservation, members of the CDF family have been comprehensively identified in prokaryotes, archaea, and eukaryotes (including plants) (Montanini et al., 2007). Based on substrate specificity for divalent metal ions, the CDF family is classified into three major subfamilies: Mn-, Zn/Fe-, and Zn-CDFs. Most CDF proteins are characterised by the presence of six transmembrane domains (TMDs) and a conserved C-terminal cation efflux domain that extends into the cytoplasm (Montanini et al., 2007).

In plants, CDF transporters are typically referred to as metal tolerance proteins (MTPs). Plant MTPs function as H^+^/metal-ion antiporters driven by proton gradients (Yang et al., 2022). These transporters confer metal tolerance by compartmentalising excess or toxic metal ions (e.g., Cd^2+^) into vacuolar compartments. In addition, MTPs mediate the transport of indispensable metal ions, such as Zn^2+^, Mn^2+^, Fe^2+^, and Co^2+^, which is crucial for promoting plant growth and development (Haney et al., 2005). The identification of *Arabidopsis thaliana* MTP1 (AtMTP1) marked the first report of an MTP protein in plants (van der Zaal et al., 1999). Transgenic plants overexpressing *AtMTP1* exhibited enhanced resistance to Zn^2+^-induced stress, along with significantly elevated zinc ion concentrations in root tissues (Desbrosses-Fonrouge et al., 2005). MTP family members play essential roles in various plant species by mediating the transport of diverse divalent metal ions. For instance, in response to heavy metal stress from elevated Zn^2+^ and Cd^2+^ levels, the transcript levels of *MTP1*, *MTP3*, and *MTP4* in *Citrus sinensis* were markedly induced in root or foliar tissues. *AtMTP11* enhances Mn^2+^ tolerance in both yeast and *A. thaliana* (Delhaize et al., 2007). Heterologous expression of *Populus trichocarpa MTP8.1*, *MTP9*, and *MTP10.4* in yeast cells demonstrated their specific roles in Mn^2+^ translocation, whereas *MTP6* showed broad substrate specificity for Co^2+^, Fe^2+^, and Mn^2+^ ions (Gao et al., 2020). Collectively, these findings underscore the critical functions of the *MTP* gene family in mediating plant responses to heavy metal stress.

With the increasing availability of genome sequences for a wide range of plant species, numerous MTP proteins have been identified and verified in species including *A. thaliana* (van der Zaal et al., 1999), *Triticum aestivum* (Vatansever et al., 2017), sweet orange (Fu et al., 2017), grape (Shirazi et al., 2019), tobacco (Liu et al., 2019), rice (Ram et al., 2019), *P. trichocarpa* (Gao et al., 2020), potato (Li et al., 2021), tomato (El-Sappah et al, 2021), *Quercus dentata* (Jiang et al., 2024), and *Nelumbo nucifera* (Hu et al., 2025). *Coptis chinensis* Franch. is a well-known traditional medicinal plant in China. Its dried rhizome is noted for its strong antibacterial and antifungal activities and is used in traditional medicine for clearing heat, eliminating dampness, and detoxifying (Liu et al., 2022). Due to the high levels of alkaloids in its rhizomes, *C. chinensis* is often referred to as the ‘Chinese antibiotic’. However, as a perennial herb, the rhizome of *C. chinensis* has been found to accumulate at more than 1.64 µM Cd in numerous batches of commercial samples (Huang et al., 2019). The presence of such elevated Cd levels notably impacts the quality of the rhizome and poses a severe threat to the safety and efficacy of *C. chinensis*. Therefore, identifying and characterising MTP family genes in *C. chinensis* is important for understanding their roles in heavy metal detoxification in this species. With the availability of high-quality genome sequencing and assembly for *C. chinensis*, a systematic analysis of the *MTP* gene family is now feasible (Liu et al., 2021).

A comprehensive functional elucidation and systematic investigation of CcMTPs in *C. chinensis* remain unconducted. In this study, we aimed to identify *CcMTPs* in the *C. chinensis* genome and comprehensively investigate the *MTP* gene family, including phylogenetic relationships, gene structure, conserved motifs, cis-regulatory elements, protein-protein interaction network, and expression patterns in different tissues, in response to Cd toxicity. The rationale was to elucidate novel perspectives on the functions of *CcMTP* genes in response to Cd stress in *C. chinensis*.

## Materials and methods

### Plant materials and Cd treatments

Four-year-old *C. chinensis* samples were collected from Gaochuan Township, Anzhou District, Mianyang City, China (31°37′49.8″N, 104°12′5.7″E). Seedlings of *C. chinensis* were cultivated in a greenhouse maintained at 22°C under 16-h day/8-h night cycles with 70% relative humidity at the Key Laboratory of Plant Genetics and Molecular Breeding for 7d. Based on previous research, the seedlings were washed and then transferred into aerated containers filled with half-strength Hoagland’s solution supplemented with 100μM CdCl_2_ for Cd treatment (Huang et al., 2019). After exposure to Cd for 0h, 6h, 12h, 24h, and 48h, the roots and leaves of *C. chinensis* seedlings were harvested for analysis of the expression patterns of the *CcMTP* gene family. Samples were immediately frozen in liquid nitrogen and stored at –80°C.

### Identification of *CcMTP* genes

The genome sequence of *C. chinensis* was retrieved from the NCBI database (https://www.ncbi.nlm.nih.gov/datasets/genome/GCA_015680905.1/). To identify all *MTP* genes in *C. chinensis*, the HMM profile of the cation efflux domain (PF01545) was downloaded from the Pfam protein family database (http://pfam.xfam.org) and used to screen for potential *MTP* genes within the *C. chinensis* genome using HMMER 3.2.1 (http://hmmer.janelia.org/) (Prakash et al., 2017). After removing redundant sequences, the putative CcMTP protein sequences were submitted to the NCBI Conserved Domains Database (https://www.ncbi.nlm.nih.gov/cdd/), SMART database (http://smart.embl.de/) and Pfam for additional confirmation of conserved domains (Marchler-Bauer et al., 2017; Letunic et al., 2021). To further validate the completeness of our identification, we conducted a BLASTP search against the *C. chinensis* proteome using experimentally characterised MTP protein sequences from *A. thaliana* and *Oryza sativa* as queries. This independent analysis confirmed that all candidates identified via the HMM-based approach were consistently recovered, with no additional *CcMTP* homologs detected.

### Chromosomal localisation and duplication analysis of *CcMTPs* in *C. chinensis*

The chromosomal location data of *CcMTPs* were retrieved from the assembled *C. chinensis* genomic repository. These spatial arrangements and gene duplication events were subsequently subjected to graphical representation through TBtools software (Chen et al., 2020). The rates of synonymous (Ks) and non-synonymous (Ka) substitutions per site for duplicated gene pairs were also calculated using TBtools (Chen et al., 2020).

### Phylogenetic analysis of CcMTPs

Twenty-five CcMTP protein sequences were obtained from NCBI. Subsequently, protein sequences of the *MTP* gene family from *Arabidopsis* and rice were used to construct a phylogenetic tree using MEGA version 10.0, based on the maximum likelihood method with bootstrap values calculated from 1000 replicates (Tamura et al., 2011). The phylogenetic tree was annotated and visualised using EvolView 2.0 server (https://www.evolgenius.info/evolview-v2/).

### Gene structure, conserved motifs, cis-regulatory elements, and protein modelling of CcMTPs

The physicochemical properties of the confirmed CcMTP proteins, including molecular weight (MW) and theoretical isoelectric point (pI), were calculated using the ExPASy server (https://web.expasy.org/protparam/). TMHMM and WoLF PSORT were used to predict transmembrane domains and subcellular localisation, respectively (https://wolfpsort.hgc.jp/) (Krogh et al., 2001; Yu et al., 2006). Conserved motifs within CcMTP proteins were identified using the MEME server (http://meme-suite.org/tools/meme) with default parameters, specifying up to 10 motifs per gene and motif widths between 15 and 40 amino acids. Conserved domains of CcMTP sequences were further confirmed using InterPro (https://www.ebi.ac.uk/interpro/). Exon-intron structures of *CcMTP*s were analysed using the GSDS 2.0 online tool (http://gsds.gao-lab.org/). The 2000 bp upstream regions of the 25 identified *CcMTP*s were extracted as promoter sequences, and related cis-regulatory elements were identified using the PLACE database (http://bioinformatics.psb.ugent.be/webtools/plantcare/html/). Computational structural modelling of CcMTP proteins was performed using the intensive mode of the Phyre2 webserver (http://www.sbg.bio.ic.ac.uk/phyre2/) (Kelley et al., 2015).

### Sequence similarity network (SSN) analysis

Protein sequences of MTPs from *A. thaliana*, *O. sativa*, and *C. chinensis* were subjected to pairwise alignment using the NCBI BLASTP algorithm with default parameters (e-value ≤ 1e–5). Gene pairs with sequence similarity greater than 46% were retained. An SSN was constructed by EFI-EST (Oberg et al., 2023) and visualised using Cytoscape software (v3.10.3). In the network, nodes represent individual protein sequences, and edges represent significant BLASTP alignments meeting a significance threshold (e-value ≤1e-5), and topological organisation was optimised via Cytoscape force-directed layout (Shannon et al., 2003).

### Expression patterns of *CcMTPs* in *C. chinensis*

Gene-specific oligonucleotide primers were designed and optimised using Primer3 software (**Supplementary Table S2**) for RT-qPCR analysis. Total RNA was extracted from the 12 samples described previously using the HiPure Total RNA Plus Kit (Magen, R411103). First-strand cDNA synthesis was performed using the PrimeScript RT reagent kit with gDNA Eraser (TaKaRa, RR047A), following the manufacturer’s protocol. The synthesised cDNA served as a template for qPCR, which was performed using the CFX96™ Real-Time PCR Detection System (Bio-Rad, USA) with SYBR Premix Ex Taq™ (Tli RNaseH Plus, Takara Bio Inc.). The amplification protocol was as follows: initial denaturation at 95°C for 3min, followed by 40 cycles of 95°C for 10s and 60°C for 30s. All reactions were performed in triplicate under identical conditions. The fold changes in RNA transcripts were computed using the 2^−ΔΔCt^ method (Livak and Schmittgen, 2001), with the 18S rRNA-encoding gene (DQ406855) in *C. chinensis* used as the internal reference (Liu et al., 2022) (**Supplementary Table S2**).

### Yeast complementation assay

To validate the functional roles of *CcMTP*s, the full-length coding sequences (CDS) of *CcMTPs* were amplified using Phanta Max Super-Fidelity DNA Polymerase (Vazyme, China). Following sequencing confirmation, PCR amplicons of three target genes (*CcMTP11*, *CcMTP16*, and *CcMTP24*) were directionally cloned into *Hind* III/*Sac* I restriction sites of the pYES2 yeast expression vector, generating three recombinant plasmids (pYES2-*CcMTP11*, pYES2-*CcMTP16*, and pYES2-*CcMTP24*) along with an empty vector control. These plasmids were transformed into the Cd -hypersensitive *Saccharomyces cerevisiae* mutant strain Δ*ycf1* via lithium acetate-mediated transformation for heterologous complementation analysis. Primary transformants were selected on uracil dropout (SD-Ura) agar medium. Cultures were grown at 28°C with orbital shaking (225 rpm) at 28°C until reaching mid-exponential phase (OD_600_ = 1.0 ± 0.05) and serially diluted 10-fold (10^-1^, 10^-2^, 10^-3^). A 50-μL aliquot from each dilution was spotted onto SGR–Ura plates supplemented with various heavy metals: Cd^2+^ (0, 10, 30, 55μM), Mn^2+^ (0, 20, 30, 40mM), Fe^2+^ (0, 40, 60, 65mM), and Zn^2+^ (0, 8, 10, 12mM). Plates were incubated at 30°C for 3–5 d before imaging.

To assess Cd -induced growth inhibition, yeast cells were cultured in 20mL SGR–Ura medium to an OD_600_ = 0.2 and treated with 35μM CdCl_2_. OD_600_ was measured at 6-h intervals to generate a growth curve of the transformants. To analyse Cd accumulation within yeast cells, yeast cells were propagated in 20 mL of SGR-Ura medium until the OD_600_ reached 0.2, followed by a 48-h exposure to 35 μM CdCl_2_ under standard culture conditions. The yeast cells were then harvested and washed three times with distilled water. Subsequently, they were lyophilised for 48h at 85°C to constant weight, and digested in 5mL nitric acid. Intracellular metal content was determined using inductively coupled plasma-optical emission spectrometry (ICP-OES 7000, Thermo Fisher Scientific, New York, USA).

### Statistical analysis

Statistical analyses of numerical data were performed using one-way analysis of variance (ANOVA) and independent-sample *t*-tests, followed by *post-hoc* Duncan tests (*p* < 0.01) and least significant difference (LSD) comparisons. All computations were executed via the SPSS, version 23.

## Results

### Sequence characteristics of CcMTP proteins

Using 12 AtMTP and 10 OsMTP protein sequences as the queries, a total of 25 MTP proteins were identified in the *C. chinensis* genome. According to the sequence analysis, multiple distinct characteristics of the CcMTPs were elucidated (**Table 1**; **Supplementary Figure S1**): (1) ORF lengths of *CcMTP*s were determined to span 300–1572 base pairs (*CcMTP1* to *CcMTP25*), encoding 99–523 amino acid residues. Predicted molecular weights (MWs) ranged from 11.4 to 58.6 kDa, and isoelectric points (pI) ranged from 4.72 to 9.05 across the CcMTP family. (2) Most of CcMTPs were predicted to contain two to six transmembrane domains (TMDs), except for CcMTP1, CcMTP18, and CcMTP21, which harboured ten, seven, and eight TMDs, respectively. (3) Subcellular localisation by Wolfpsort predicted that 24 of the 25 proteins localised in both the plasma membrane and tonoplast, with only CcMTP11 predicted to be located in the Golgi apparatus. (4) Multiple sequence alignment revealed that three distinct subfamilies (Zn-, Zn/Fe-, Mn-MTPs) exhibited low sequence homology (**Supplementary Figure S1**). The *CcMTP* members classified within the Zn-MTP and Zn/Fe-MTP clades exhibited a conserved HXXXD (X = variable residue) signature sequence spanning both TMD-I and TMD-V. Conversely, the Mn-MTP subgroup was characterised by a divergent DXXXD motif localised in these equivalent transmembrane regions, suggesting evolutionary adaptation for differential metal ion selectivity (**Supplementary Figure S1**).

**Table 1:** Physicochemical characterisation and subcellular localisation profiling of 25 metal tolerance proteins (MTPs) identified in *Coptis chinensis*.

Gene	Gene ID	Chromosome location	Strand	ORF (bp)	Amino acid (aa)	MW (Da)	Pl	Exon_num	Intron_num	TMD_num	Subcellular localization
*CcMTP1*	gene-IFM89_000800	Ch1:24896634.24898849	reverse	1572	523	58615.2	6.59	3	2	10	plasma membrane
*CcMTP2*	gene-IFM89_016928	Ch1:48898364.48900678	forward	363	120	12671.5	6.68	4	3	2	vacuolar membrane
*CcMTP3*	gene-IFM89_022165	Ch1:62001082.62008411	reverse	534	177	19177.8	6.27	4	3	3	vacuolar membrane
*CcMTP4*	gene-IFM89_030069	Ch2:51612786.51614126	forward	333	110	11773.8	9.05	3	2	2	vacuolar membrane
*CcMTP5*	gene-IFM89_015835	Ch2:66303915.66310412	forward	1443	480	52274.9	8.33	12	11	3	vacuolar membrane
*CcMTP6*	gene-IFM89_015867	Ch2:67014718.67016610	reverse	615	204	22078.4	6.49	3	2	2	plasma membrane
*CcMTP7*	gene-IFM89_013972	Ch2:95026875.95028059	forward	1185	394	43071.4	6.43	1	0	6	plasma membrane
*CcMTP8*	gene-IFM89_036276	Ch3:106730287.106731555	forward	1269	422	45977.1	6.45	1	0	6	plasma membrane
*CcMTP9*	gene-IFM89_018594	Ch4:5571982.5585216	forward	708	235	25365.2	6.91	6	5	2	vacuolar membrane
*CcMTP10*	gene-IFM89_015722	Ch4:5622152.5623639	forward	372	123	13873.4	8.09	4	3	2	vacuolar membrane
*CcMTP11*	gene-IFM89_012967	Ch5:33038739.33040479	forward	582	193	20918.1	6.58	5	4	3	Golgi apparatus
*CcMTP12*	gene-IFM89_039219	Ch5:45379320.45384909	forward	795	264	30140.6	7.94	4	3	2	vacuolar membrane
*CcMTP13*	gene-IFM89_039220	Ch5:45388330.45394008	forward	585	194	22324.9	4.72	4	3	2	plasma membrane
*CcMTP14*	gene-IFM89_008993	Ch5:45439432.45447539	forward	1515	504	56982.1	6.55	7	6	5	plasma membrane
*CcMTP15*	gene-IFM89_024537	Ch6:13220894.13224809	reverse	930	309	34133.5	7.61	7	6	4	plasma membrane
*CcMTP16*	gene-IFM89_033964	Ch6:31794435.31800073	reverse	453	150	15929.5	6.59	5	4	2	plasma membrane
*CcMTP17*	gene-IFM89_023984	Ch6:99559185.99566522	forward	864	287	31934.7	5.9	5	4	3	plasma membrane
*CcMTP18*	gene-IFM89_016406	Ch7:984983.990093	forward	1167	388	43641.1	8.98	10	9	7	plasma membrane
*CcMTP19*	gene-IFM89_025557	Ch7:21116313.21117527	reverse	1215	404	44921.7	6.72	1	0	6	plasma membrane
*CcMTP20*	gene-IFM89_032735	Ch7:68249411.68254128	reverse	1170	389	44139.3	4.91	7	6	4	plasma membrane
*CcMTP21*	gene-IFM89_032729	Ch7:68318637.68323084	reverse	1485	494	55939.1	5.74	9	8	8	plasma membrane
*CcMTP22*	gene-IFM89_031612	Ch8:77692925.77693900	forward	624	207	22998.7	8.75	2	1	4	vacuolar membrane
*CcMTP23*	gene-IFM89_011813	Ch8:79968297.79969780	reverse	300	99	11359.3	7.35	3	2	2	vacuolar membrane
*CcMTP24*	gene-IFM89_035896	Ch9:41545531.41546893	forward	399	132	13808.2	7.51	5	4	3	plasma membrane
*CcMTP25*	gene-IFM89_031729	Ch9:52306450.52308589	forward	441	146	16659.2	6.39	4	3	2	plasma membrane

### Chromosomal localisation and synteny analysis of *CcMTPs*

**Figure 1:**
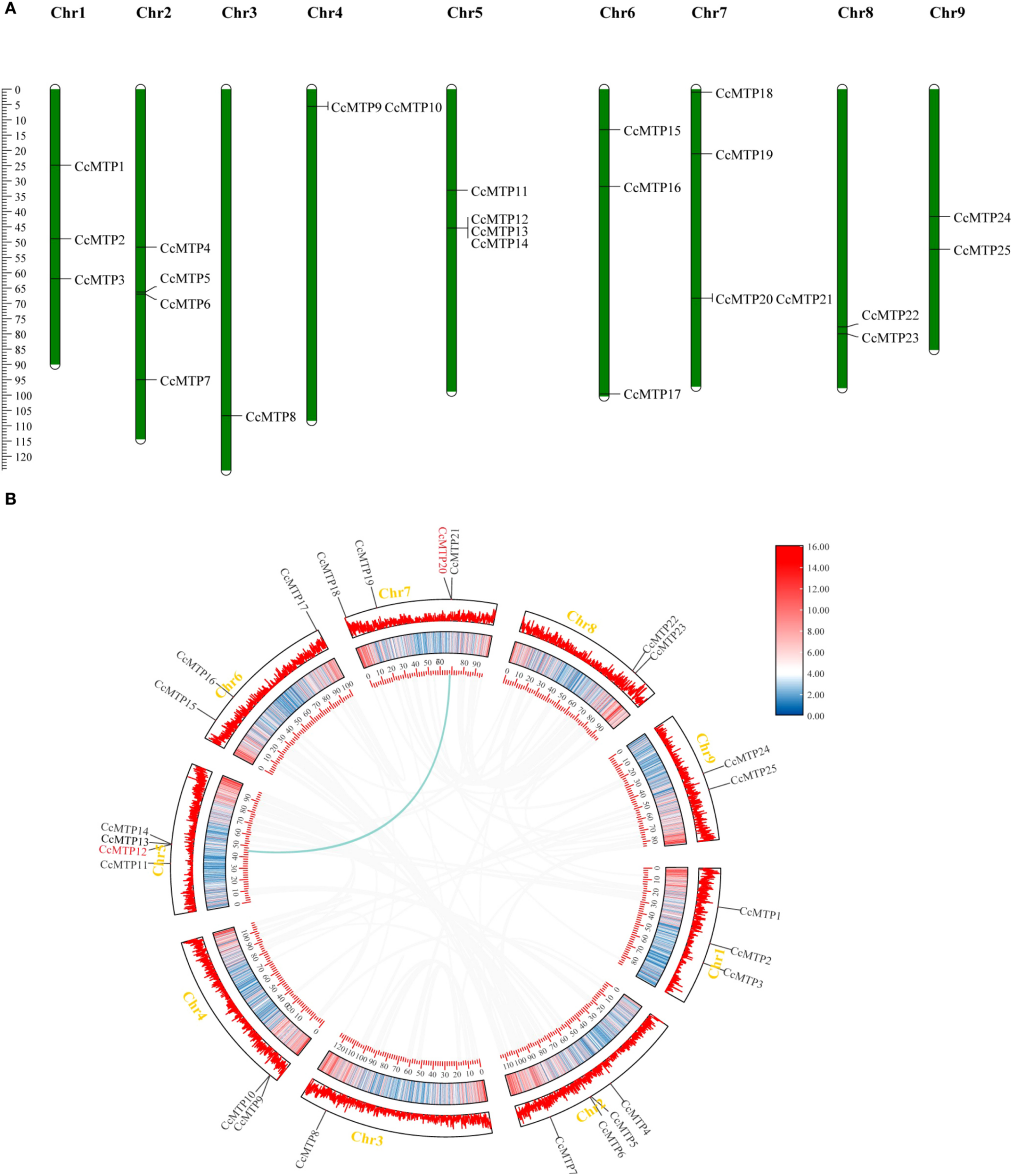
Chromosomal locations and synteny analysis of *CcMTP* genes. **(A)** Chromosomal locations of *CcMTP* genes in *Coptis chinensis*. **(B)** The chromosomal relationships of *CcMTPs* in *C*. *chinensis* were identified using multiple collinear scanning toolkits (specifically the one Step MCScanX super Fast module in TBtools 2.0) and visualised with TBtools. Gray lines denote collinear modules within the *C*. *chinensis* genome, whereas blue lines highlight *MTP* collinear genes.

To characterise the chromosomal distribution of *C. chinensis MTP* genes, we mapped the genomic locations of the 25 *CcMTP* genes across the *C. chinensis* genome (**Figure 1A**). This study demonstrated a heterogeneous distribution pattern across the nine chromosomes. Chr2, Chr5, and Chr7 harboured the highest number of *CcMTP* genes (4) CcMTP genes, with Chr1 and Chr6 following closely behind, each containing three *CcMTP* genes. Chr4, Chr8, and Chr9 each harboured two *CcMTP* genes, whereas the lowest number (one *CcMTP* gene) was identified on Chr3.

In this study, we employed the TBtools software to investigate gene duplication events within the *CcMTP* gene family. Genomic analyses revealed that the *CcMTP* gene family generated a single segmental duplication event involving the *CcMTP12* and *CcMTP20* gene pair, as evidenced by phylogenetic and syntenic conservation patterns (**Figure 1B**). This isolated chromosomal duplication event likely represents an evolutionary mechanism driving the functional diversification of metal transport systems in *C. chinensis*. To investigate the evolutionary dynamics and selective pressures influencing the *CcMTP* gene family, the values of Ka, Ks, and the Ka/Ks ratio were calculated in the *C. chinensis* genome. The duplicated *CcMTP* gene pairs exhibited a Ka/Ks ratio of 0.69 (**Supplementary Table S3**), suggesting that *MTP* family genes in *C. chinensis* may have undergone selective pressure during their evolutionary history.

### Phylogenetic relationships and classification of *MTP* genes

**Figure 2:**
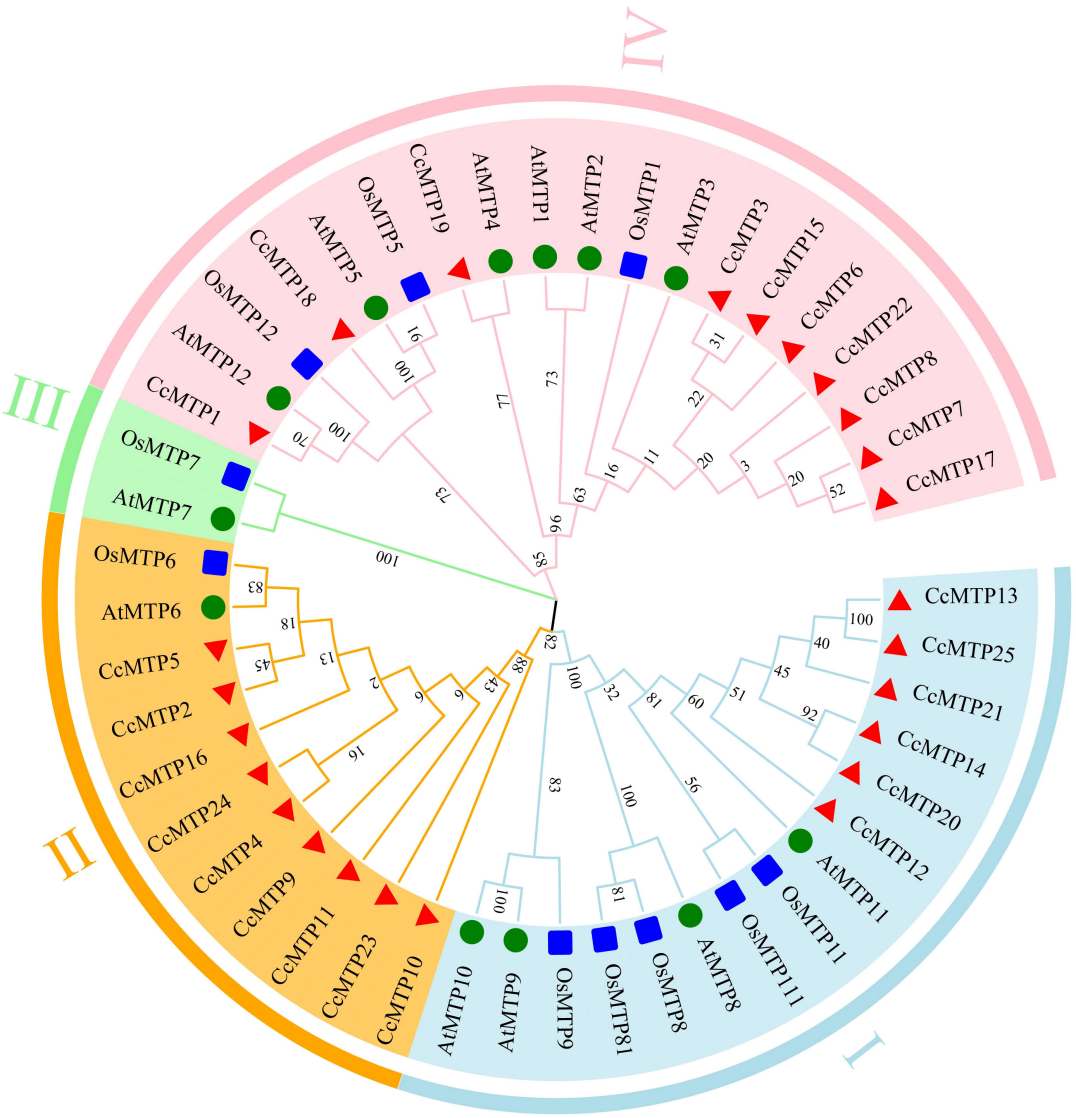
Phylogenetic analysis of MTP proteins from different species. Species abbreviations: At, *Arabidopsis thaliana*; Os, *Oryza sativa*; Cc, *Coptis chinensis*.

To investigate the evolutionary relationships of the *MTP* gene family, phylogenetic analyses were conducted using the maximum likelihood (ML) method (**Figure 2**). A phylogenetic tree was constructed using MEGA X following multiple sequence alignment of 25 C*. chinensis* MTP proteins (CcMTPs), 12 A*. thaliana* MTP proteins (AtMTPs), and 10 rice MTP proteins (OsMTPs). CcMTP protein sequences are listed in **Supplementary Table S4**. Based on the topology of the ML phylogenetic tree, *MTP* genes from *C. chinensis*, *A. thaliana*, and rice were divided into three major clades: Zn-MTP, Zn/Fe-MTP, and Mn-MTP. The three subfamilies were resolved to comprise 19, 13, and 15 MTP proteins, respectively. Multiple sequence alignment analysis of CcMTPs and AtMTPs revealed significant sequence homology, highlighting conserved structural features among these proteins (**Supplementary Figure S1**).

### Modelling and SSN analysis of CcMTP proteins

All twenty-five CcMTPs were modelled using Phyre2 with >98% confidence in normal mode to elucidate their molecular mechanisms in *C. chinensis*. Four known structural templates, c7kzxA and c8f6fA (Cd and zinc efflux pump FieF), c8j7tF and c8j7yA (zinc transporter 7), c7y5gA and c6xpdA (zinc transporter 8), and c8f6fD (another FieF structure), were used for modelling *CcMTP1–9*, *CcMTP11–14*, *CcMTP16–22*, and *CcMTP24*, respectively. Models of c3h90D (ferrous-iron efflux pump FieF), c7ttiA (solute carrier family 12 member 4), and c8xmaB and c8zsbA (proton-coupled zinc antiporter SLC30A1) were used for the remaining five CcMTPs (**Supplementary Figure S2**; **Supplementary Table S1**).

The SSN was defined as a graph structure composed of nodes and edges, where each node represents a non-redundant protein sequence (90% identity threshold), and edges connect node pairs with >40% pairwise sequence identity (BLASTP, e-value <1e-10). The MTP family SSN comprised 47 nodes (non-redundant at 90% identity) and 520 edges (≥46% sequence identity). A total of five clusters were obtained from the SSN, with clusters 1, 2, and 3 accounting for 89% of the total nodes (**Supplementary Figure S3**). The results showed that 11 CcMTP members within the Zn-MTP subfamily clustered in cluster 2, 9 transporters classified as Zn/Fe-MTPs in cluster 3, and 5 CcMTPs within the Mn-MTP clade in cluster 1. In cluster 1, six CcMTPs and AtMTP8 exhibited sequence homology ranging from 47% to 56%, whereas *CcMTP1* shared as high as 67% sequence homology with *AtMTP12* at the amino acid level. Seven *CcMTP* genes, *CcMTP3*, *CcMTP6*, *CcMTP7*, *CcMTP8*, *CcMTP15*, *CcMTP17*, and *CcMTP19*, showing significant homology with *AtMTP1* and *AtMTP3* were co-clustered in cluster 2 (**Supplementary Figure S3**).

### Conserved motifs, domains, and structure of CcMTPs

**Figure 3:**
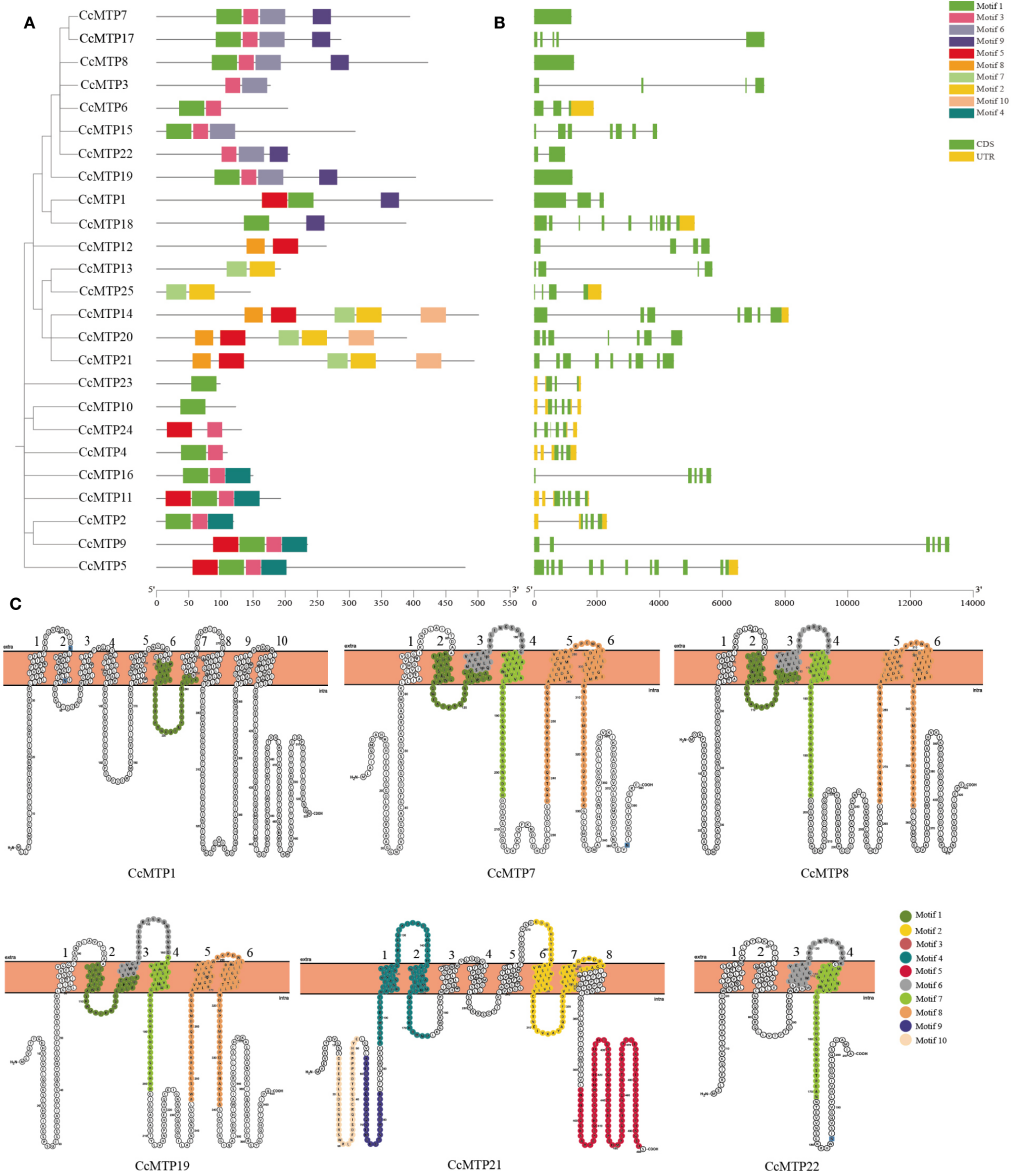
Annotation of the *CcMTP* gene family in *C*. *chinensis* including comprehensive analysis of conserved motifs, exon–intron organisation, and transmembrane helix (TM) architecture. **(A)** Phylogenetic analysis and motif characterisation of *CcMTP* gene family. Conserved motifs are distinguished by distinct colour coding across the phylogenetic tree. **(B)** Gene structures of *CcMTPs*. Exons, untranslated regions (UTRs), and introns are represented by yellow boxes, green boxes, and connecting lines, respectively. The dimensions of boxes and lines are proportional to the corresponding gene lengths. **(C)** Conserved motif arrangement across transmembrane helices (TMs). Transmembrane structures are labelled with black numbers, whereas distinct colour coding shows differently conserved motifs.

To acquire a more in-depth understanding of the structural properties of CcMTPs in *C. chinensis*, conserved motif analysis was performed using the MEME server to identify 10 evolutionarily conserved protein motifs (**Supplementary Figure S4**). The 25 *CcMTP* genes exhibited motif counts ranging from 1 to 5, demonstrating quantitative heterogeneity in conserved domain architectures (**Figure 3A**). Motif composition analysis showed that most genes (n = 20) harboured 2–4 motifs, whereas two genes (*CcMTP10* and *CcMTP23*) harboured only a single motif. Three genes (*CcMTP14*, *CcMTP20*, and *CcMTP21*) possessed five motifs. Notably, tandem duplication of motif 1 was detected in 64% of the analysed CcMTP proteins, whereas duplication was absent in *CcMTP3*, *CcMTP12*, *CcMTP13*, *CcMTP14*, *CcMTP20*, *CcMTP21*, *CcMTP22*, *CcMTP24*, and *CcMTP25*. Additionally, *CcMTP10* and *CcMTP23* exclusively contained motif 1, suggesting that motif 1 may serve as a core functional element, potentially mediating key substrate recognition activities involved in metal transport.

Notably, phylogenetic subgrouping revealed that members within the same clade demonstrated significantly similar conserved motif distribution patterns, whereas members from different clades displayed marked divergence in motif architecture (**Figure 3A**). This observation supports the hypothesis that phylogenetic clustering within the same clade correlates with functional homology, as reflected by the conserved motif arrangements among closely related members. A systematic investigation was conducted to examine the topological distribution of conserved structural motifs within transmembrane α-helices in six representative MTP family members (CcMTP1, CcMTP7, CcMTP8, CcMTP19, CcMTP21, and CcMTP22), with particular emphasis on their spatial arrangement across helical domains (**Figure 3C**).

To assess this further, domain architecture analysis of the CcMTP proteins was performed. The results showed that all CcMTP proteins contained the cation efflux domain. In contrast, the zinc transporter domain was exclusively identified in CcMTP5, CcMTP14, CcMTP20, and CcMTP21 (**Supplementary Figure S5**).

To further investigate the genomic organisation of *CcMTPs*, a comprehensive analysis of exon-intron structure was systematically performed via comparison of genomic DNA and CDS sequences (**Figure 3B**). Genomic characterisation of the *CcMTP* gene family revealed a stable exon number distribution from 1 to 12 across all members. The *CcMTP* gene family exhibited a distinct distribution pattern, with 10 members (the majority) harbouring 3–4 exons, whereas five genes (CcMTP22, *CcMTP9*, *CcMTP21*, *CcMTP18*, and *CcMTP5*) represented the extremes with 2, 6, 9, 10, and 12 exons, respectively. In contrast to the conserved motif patterns, no significant congruence was observed between phylogenetic grouping and exon-intron architecture among *CcMTP* genes within the same clade.

### Characterisation of cis-acting elements in promoters of *CcMTP* genes

**Figure 4:**
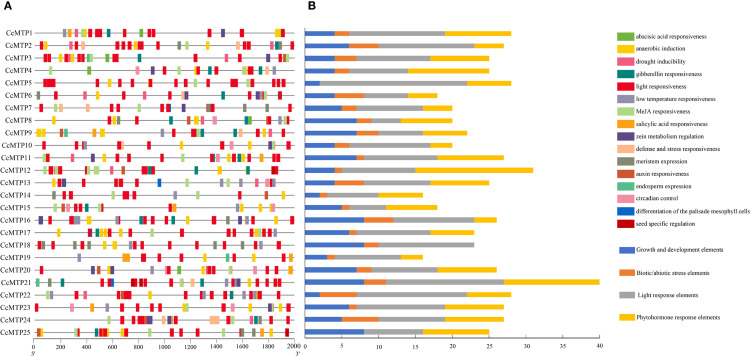
Cis-acting elements analysis of *CcMTPs* family promoters. **(A)** Distribution of cis-acting elements. **(B)** The statistics of cis-acting elements of each *CcMTP* gene.

To characterise the cis-regulatory elements within the promoters of *CcMTP* genes, 2000 bp upstream sequences from the translation start site were computationally analysed for all family members. A total of 16 major cis-acting elements were identified across the 25 *CcMTP* gene promoters (**Figure 4**). These elements were functionally categorised into four major regulatory pathways: growth and development, biotic/abiotic stress response, light responsiveness, and phytohormone responsiveness. Promoter analysis revealed that *CcMTP* gene promoters were particularly enriched in elements associated with light and biotic/abiotic stress responses, including motifs for light responsiveness, drought inducibility, low temperature responsiveness, and general defence and stress response. In addition to the major categories, *CcMTPs* gene regulatory regions comprised a small subset of elements related to phytohormone response and growth and development responsiveness, such as those responsive to abscisic acid, MeJA, and salicylic acid, along with circadian control elements.

### qRT-PCR analysis of *CcMTP* genes in different tissues under Cd^2+^ stress

**Figure 5:**
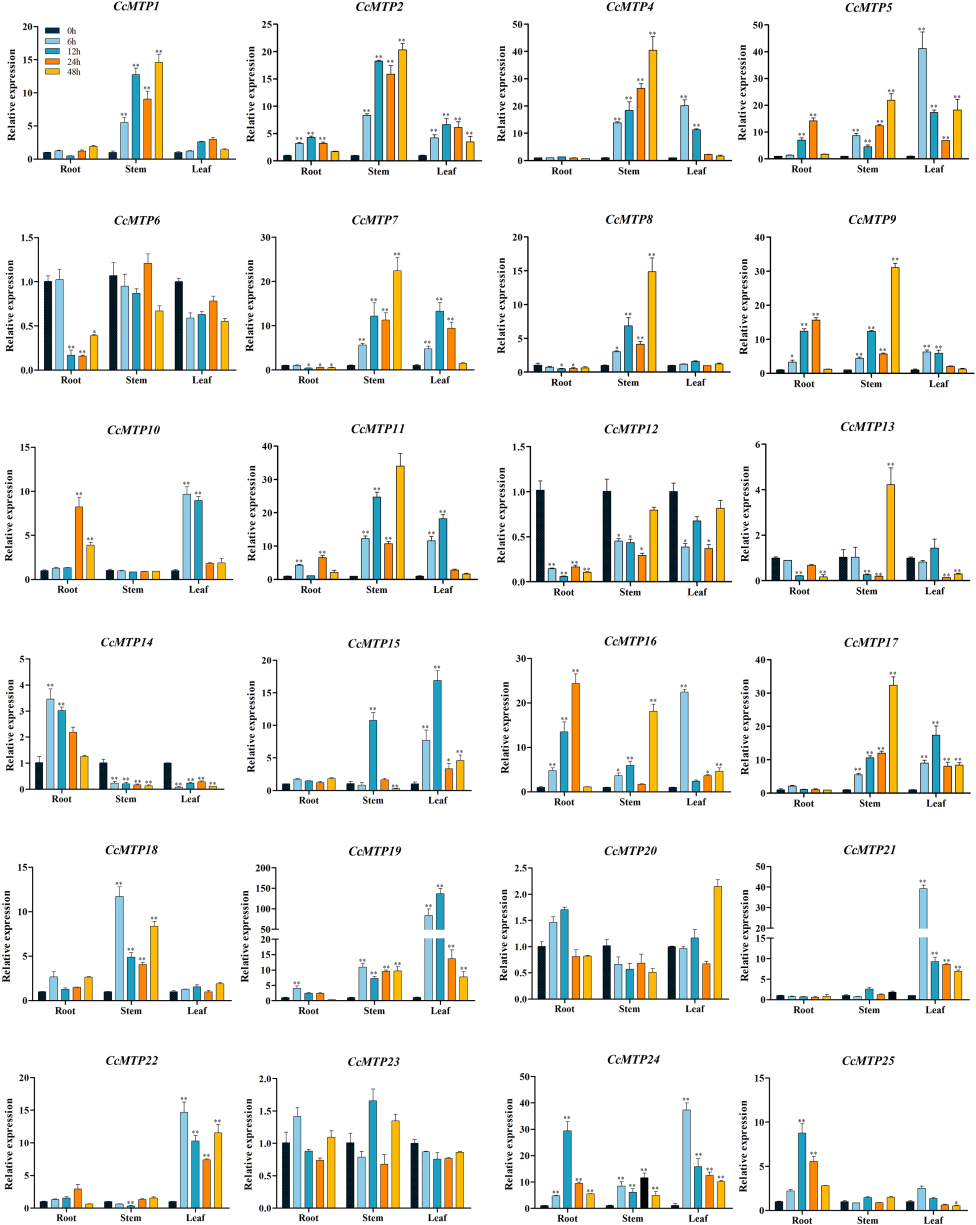
Relative expression levels of *CcMTP* genes under Cd stress in different tissues. Asterisks indicate significant differences from the control by one-way ANOVA and *t*-test followed by an independent-samples Dunnett’s *post hoc* test (*p ≤ 0.05; **p ≤ 0.01).

The relative expression patterns of 24 *CcMTP* genes under Cd^2+^ stress at 0, 6, 12, 24, and 48 h were assessed using qRT-PCR (**Figure 5**). *CcMTP3* was excluded because of unsuccessful primer design. The *CcMTP* genes exhibited diverse expression responses to Cd^2+^ exposure across roots, stems, and leaves.

In roots, Cd^2+^ increased the expression of 11 *CcMTPs* (*CcMTP2*, *CcMTP5*, *CcMTP6*, *CcMTP9*, *CcMTP10*, *CcMTP11*, *CcMTP14*, *CcMTP16*, *CcMTP19*, *CcMTP24*, and *CcMTP25*); however, it repressed the expression of five *CcMTPs* (*CcMTP7*, *CcMTP8*, *CcMTP12*, *CcMTP13*, and *CcMTP17*). In stems, Cd^2+^ markedly enhanced the expression of 62.5% of *CcMTP genes*, except for *CcMTP6*, *CcMTP10*, *CcMTP20*, *CcMTP21*, and *CcMTP23*. In contrast, it remarkably suppressed the expression of *CcMTP12*, *CcMTP13*, *CcMTP14*, and *CcMTP22*. Notably, the transcript level of most of *CcMTPs* reached the peak at 48 h after Cd treatment. For instance, the expression levels of *CcMTP4* in stems reached their highest at 48 h, which were 40.5-fold higher than those in the control. In leaves, 58.3% of the *CcMTP* genes were remarkably upregulated under Cd treatment, except for *CcMTP1*, *CcMTP6*, *CcMTP8*, *CcMTP18*, *CcMTP20*, *CcMTP23*, and *CcMTP25*, whereas the expression levels of *CcMTP12*, *CcMTP13*, and *CcMTP14* were downregulated. Excess Cd significantly upregulated the expression levels of *CcMTP2*, *CcMTP5*, *CcMTP9*, *CcMTP11*, *CcMTP16*, *CcMTP19*, and *CcMTP24*. In contrast, it substantially downregulated the expression levels of *CcMTP12* and *CcMTP13* in three tissues. Given that smaller gene size facilitates PCR amplification, efficient transformation into yeast for functional characterisation, as well as proper expression and folding, *CcMTP11*, *CcMTP16*, and *CcMTP24* were selected for subsequent functional validation in this study. *CcMTP11*, *CcMTP16*, and *CcMTP24* were selected for subsequent functional validation in this study.

### Functional analysis of CcMTP proteins in yeast

**Figure 6:**
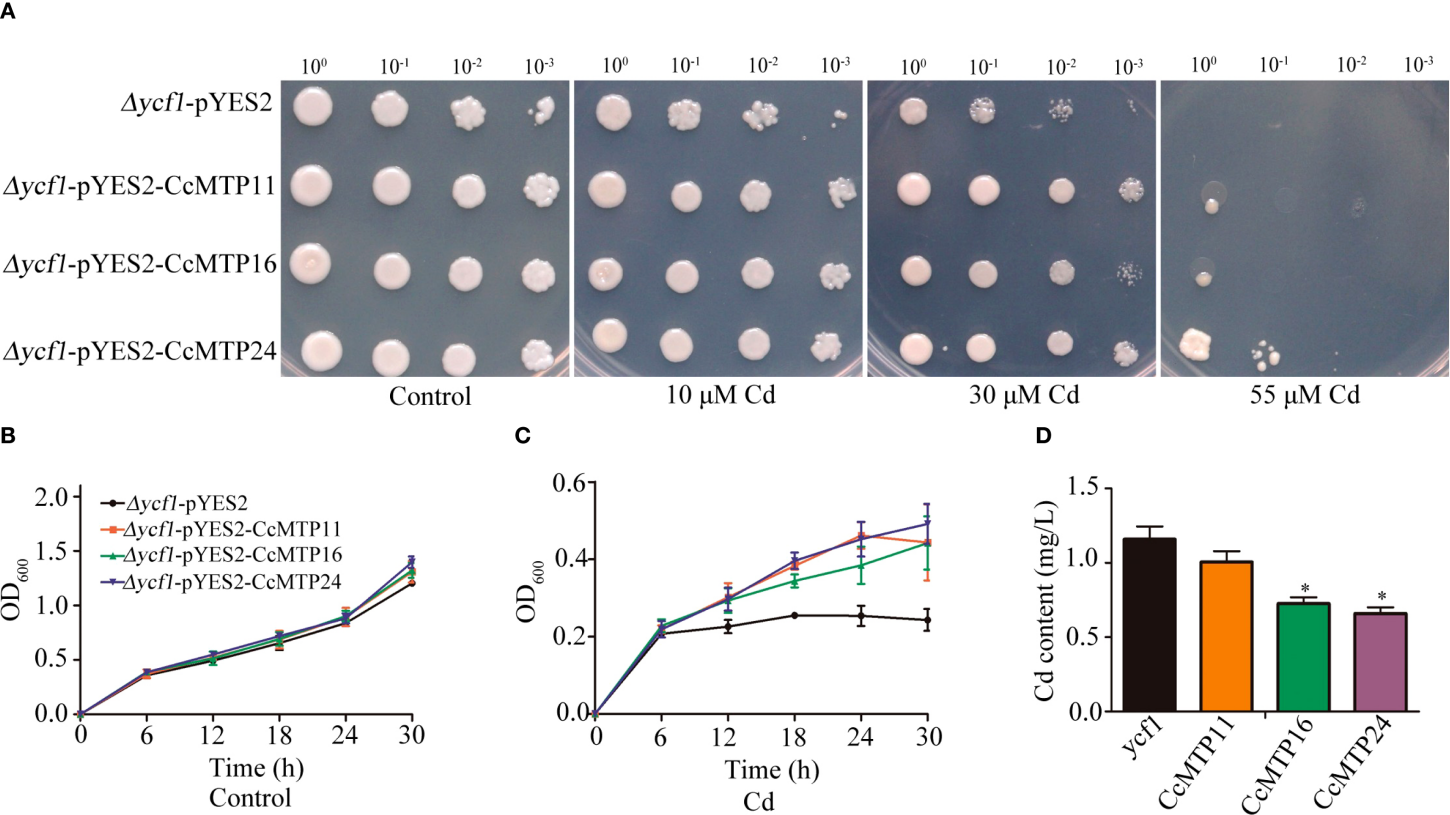
Functional analysis of CcMTP11, CcMTP16, and CcMTP24 in the yeast cells. **(A)** △ycf1 mutant harbouring empty vectors or recombinant plasmids encoding CcMTP11/CcMTP16/CcMTP24 were cultured on SGR-Ura solid medium supplemented with 0, 10, 30 or 55 µM Cd for 3 days. **(B, C)** Growth dynamics of yeast cells at different time intervals in the absence **(B)** or presence of CdCl₂ **(C)**. Yeast cultures were adjusted to an OD₆₀₀ of 0.2, followed by the addition of CdCl₂ to achieve a final concentration of 35 µM in the medium. Samples were harvested and OD₆₀₀ was measured at 6-hour intervals thereafter. **(D)** The content of Cd in yeast cells. ycf1, CcMTP11, CcMTP16, and CcMTP24 represented △ycf1 mutant harbouring empty vectors or recombinant plasmids encoding CcMTP11, CcMTP16, and CcMTP24. Asterisks indicate significant differences from the control by one-way ANOVA and t-test followed by an independent-samples Dunnett’s post hoc test (*, p ≤ 0.05).

Based on the expression patterns of *CcMTP* genes in Cd-treated *C. chinensis* plants, *CcMTP11, CcMTP16*, and *CcMTP24*, which exhibited high expression levels in roots, stems, and leaves, were selected as the candidate genes for functional analysis in yeast. The yeast mutant Δ*ycf1*, deficient in yeast Cd factor 1, was transformed with the yeast expression vector pYES2 carrying *CcMTP11, CcMTP16*, or *CcMTP24*, as well as with the empty vector as a control. When cultured on standard SGR-Ura medium without Cd, or with 10 µM and 30 µM Cd, the growth phenotypes of yeast strains overexpressing *CcMTPs* were indistinguishable from those harbouring the empty vector. However, on medium supplemented with 55 μM Cd, *CcMTP24* significantly enhanced the Cd tolerance of the Δ*ycf1* mutant, whereas *CcMTP11* and *CcMTP16* marginally improved Cd tolerance (**Figure 6A**).

Growth curve analysis further confirmed that yeast strains transformed with *CcMTP11, CcMTP16*, and *CcMTP24* exhibited significantly higher growth rates under Cd stress compared with the Δ*ycf1*-pYES2 control (**Figure 6C**). In contrast, no significant growth differences were observed under normal conditions (**Figure 6B**). Notably, after 48 h of exposure to 35 µM Cd, all three transformants accumulated lower Cd levels than the Δ*ycf1* control, with *CcMTP16* and *CcMTP24* showing especially reduced Cd accumulation (**Figure 6D**). These results suggest that *CcMTP24* may mediate Cd detoxification by facilitating its efflux into the extracellular medium.

**Figure 7:**
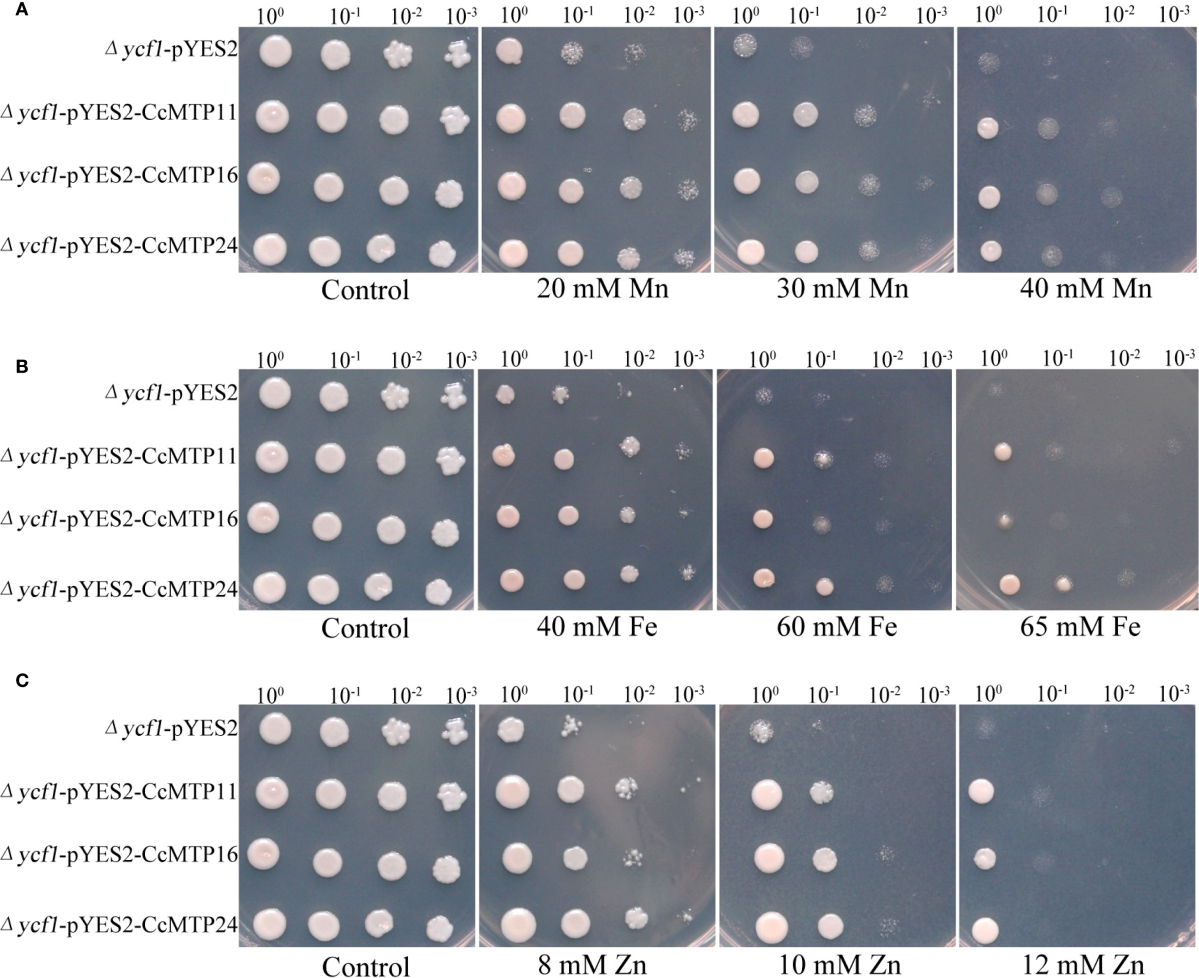
Heterologous expression-mediated tolerance of yeast to Mn, Zn, and Fe conferred by *CcMTP11*, *CcMTP16*, and *CcMTP24*. Yeast cells were subjected to transformation with either the empty vector pYES2 or *CcMTP11*, *CcMTP16*, and *CcMTP24*, then inoculated onto SGR-Ura solid medium amended with 0, 20, 30, 40 mM MnCl_2_ **(A)**, 0, 40, 60, 65 mM FeSO_4_ **(B)**; 0, 8, 10–12 mM ZnSO_4_ **(C)**.

Additionally, we investigated whether *CcMTP11, CcMTP16*, and *CcMTP24* conferred tolerance to other heavy metals, such as Mn, Zn, and Fe. When expressed in yeast, *CcMTP11* and *CcMTP24* conferred tolerance to Mn, Zn, and Fe, whereas *CcMTP16* enhanced tolerance only to Mn and Zn (**Figure 7**). These findings demonstrate that the heterologous expression of *CcMTP11, CcMTP16*, and *CcMTP24* in yeast enhances cellular tolerance to various heavy metals.

## Discussion

*MTP* genes exhibit ubiquitous distribution across the plant kingdom and encode membrane-localised divalent cation transporters that function in mediating tolerance to and transmembrane transport of diverse heavy metals, potentially playing critical roles in maintaining plant mineral nutrition homeostasis and enhancing resistance to metal-induced stresses (Liu et al., 2024; Li et al., 2024; Hu et al., 2025).

In this study, 25 *CcMTP* genes were identified and classified into four groups in conjunction with orthologs from *A. thaliana* and rice and further assigned to three major substrate-specific clades: Zn-MTPs, Zn/Fe-MTPs, and Mn-MTPs (**Figure 2**). These findings are consistent with the results reported in peanut (Wang et al., 2022), tulip (Lu et al., 2024), *Q. dentata* (Jiang et al., 2024), *T. aestivum* (Vatansever et al., 2017), *Brassica napus* (Xie et al., 2022), and *Nicotiana tabacum* (Liu et al., 2019), indicating that *CcMTPs* may exhibit functional similarities to their orthologs in these species. Among plant species with characterised *MTP* gene families, *C. chinensis* harbours fewer *MTP* genes than *B. napus* (33 MTPs), *T. aestivum* (33 MTPs), and *N. tabacum* (26 MTPs), whereas most other species contain 10–12 MTP members (Xie et al., 2022; Vatansever et al., 2017; Liu et al., 2019; Zhao et al., 2024).

Phylogenetic analysis revealed that the CcMTPs cluster into three primary groups (**Figure 2**), a classification that aligns with the corresponding groupings of *A. thaliana* and rice. Most plants (e.g., tomato, potato, soybean, *Q. dentata*, and *B. napus*) contain more Mn-MTP members than Zn-MTPs (El-Sappah et al., 2021, 2023; Xie et al., 2022; Jiang et al., 2024; Li et al., 2021); however, the contrary pattern was observed in *C. chinensis* and *A. thaliana* (van der Zaal et al., 1999), where Zn-MTP members outnumber Mn-MTP members. Phylogenetic analysis further revealed that the six Mn-MTP subfamily members in *C. chinensis* cluster closely with AtMTP8, AtMTP11, OsMTP8, and OsMTP11, whereas 10 Zn-MTP members and nine additional MTPs formed a clade with MTPs from *A. thaliana* and rice (**Figure 2**). Notably, homologous genes within the same phylogenetic clade are likely to exhibit shared or analogous biological functions (Liu et al., 2023).

As previously described, while the cation efflux domain represents a characteristic feature of MTP transporters (Liu et al., 2019), to date, the biological roles of *CcMTPs* remain largely unelucidated. Nonetheless, the well-characterised functions of *MTPs* in other species (e.g., *A. thaliana* and rice) may facilitate the prediction of gene functions in *C. chinensis* via ortholog-based analysis. For instance, AtMTP8 and AtMTP11 are two Mn-MTP proteins, the functions of which, have been clearly defined. AtMTP8, a Golgi or vacuolar Mn transporter, not only contributes to the protection of plants from Mn toxicity but also is involved in the distribution of Fe and Mn in seeds (Eroglu et al., 2016, 2017; Chu et al., 2017). AtMTP11, localised in the prevacuolar compartment, exhibits Mn²^+^-specific transport activity and plays an essential role in Mn transport and tolerance (Delhaize et al., 2007). In rice, OsMTP8.1 and OsMTP8.2 are tonoplast-localised Mn transporters involved in Mn transport (Tsunemitsu et al., 2018). Compared with AtMTP11, OsMTP11 not only confers Mn tolerance but also actively sequesters Cd into leaf vascular parenchyma cells, preventing its translocation to grains (Farthing et al., 2017; Liu et al., 2024). These findings suggest a potential role for CcMTP proteins in regulating metal ion efflux and maintaining metal homeostasis, highlighting their utility as valuable genetic resources for breeding *C. chinensis* with low Cd.

Notably, three Zn-MTP genes (*CcMTP1*, *CcMTP8*, and *CcMTP19*) exhibit coding sequences composed of a single exon devoid of introns, classifying them as single-exon genes (SEGs) (Sakharkar et al., 2004). The presence of SEGs in multicellular eukaryotic genomes is of particular interest, because such genes are characteristically archetypal of prokaryotic systems (Sakharkar et al., 2004). SEGs are categorised into two distinct groups: (i) untranslated region (UTR) intron-containing SEGs (uiSEGs), which harbour introns within their UTRs; and (ii) intronless genes (IGs), which lack introns throughout the gene structure (Jorquera et al., 2018). In this study, *CcMTP1, CcMTP8*, and *CcMTP19* were identified as IGs. SEGs have been documented within the MTP family of peanut (Wang et al., 2022), tomato (El-Sappah et al., 2021), and tobacco (Liu et al., 2019). These results suggest that during the evolutionary history of *MTP* genes in *C. chinensis*, episodes of intron gain and loss occurred. Certain genes lack introns and consist of a single exon, exhibiting reduced rates of exon gain or loss due to stronger selective pressure acting on exon sequences (Harrow et al., 2006).

Notably, CcMTP1 contains 10 TMDs, more than any other CcMTPs, and was found to possess the longest protein sequence (523 amino acids) and the highest MW (58.64 kDa). Based on a phylogenetic analysis, CcMTP1 was the only MTP member in *C. chinensis* that clustered closely with AtMTP12 and OsMTP12 in a single clade (**Figure 2**), suggesting that *CcMTP1* is the homologous gene of *AtMTP12*. These findings are consistent with the characteristics of MTP12 observed in other plant species, which similarly harbour more TMDs, longer protein sequences, and higher MWs, for example, AhMTP12 (16 TMDs, 867 amino acids, and 97.04 kDa), PtrMTP12 (12 TMDs, 869 amino acids, and 97.5 kDa), and VvMTP12 (12 TMDs, 1092 amino acids, and 123.68 kDa) (Wang et al., 2022; Gao et al., 2020; Shirazi et al., 2019). These results suggest that CcMTP1 may possess unique physiological functions in *C. chinensis*.

Herein, we employed qRT-PCR to analyse the expression patterns of *MTP* genes across different tissues under Cd stress. The transcriptomic responses of *MTP* genes to the presence of their potential metal substrates exhibited notable diversity and complexity. *AtMTP1*, which encodes a tonoplast-localised zinc transporter, maintained stable expression at both transcriptional and translational levels under conditions of zinc excess (Kobae et al., 2004; Dräger et al., 2004). Similarly, the expression level of *CsMTP1* was unaffected under Zn excess, although the encoded protein abundance was significantly upregulated in cucumber under metal treatment (Migocka et al., 2015). Varying concentrations of Mn^2+^ exert minimal effects on the expression of Mn-MTP family members (*AtMTP8*, *AtMTP9*, *AtMTP10*, and *AtMTP11*), a response pattern analogous to that observed in tobacco (Delhaize et al., 2007; Liu et al., 2019). Similarly, the expression levels of Mn subfamily members in *C. chinensis* exhibited relatively minor changes compared to those in the other two subfamilies, consistent with results in tobacco and alfalfa (Liu et al., 2019; El-Sappah et al., 2021). Notably, six of nine *CcMTPs* (*CcMTP2, CcMTP5, CcMTP9, CcMTP11, CcMTP16*, and *CcMTP24*), which were simultaneously upregulated in roots, stems, and leaves of *C. chinensis*, belong to the Fe/Zn-MTP class. In contrast to other species such as *Q. dentata* (Jiang et al., 2024), tomato (El-Sappah et al., 2021), soybean (El-Sappah et al., 2023), and *P. trichocarpa* (Gao et al., 2020), where the *MTP* genes significantly responsive to cadmium stress across different tissues predominantly belong to the Zn-MTP and Mn-MTP subfamilies, it is noteworthy that in tobacco, genes from the Zn-MTP, Mn-MTP, and Fe/Zn-MTP subfamilies exhibit responses to cadmium stress in various tissues. It highlights the specific involvement of the Fe/Zn-MTP members in *C. chinensis* under Cd stress. Conversely, excessive Cd^2+^ exposure induced minimal changes in the mRNA expression levels of *CcMTP6*, *CcMTP20*, and *CcMTP23* in this study. Thus, further investigation is needed to characterise the protein-level responses of *CcMTPs* to metal ions in future research.

Currently, yeast mutants are frequently used to characterise the basic functions of genes under heavy metal stress conditions (Khan et al., 2019). *CcMTP11, CcMTP16, and CcMTP24*, members of the Fe/Zn subfamily with two or three TMDs located in the plasma membrane or Golgi (**Table 1**), were highly upregulated in roots, stems, and leaves under Cd stress (**Figure 5**). However, heterologous expression of *CcMTP24* in yeast revealed tolerance to four heavy metals, including Cd, Mn, Fe, and Zn (**Figures 6**, **7**). Comparatively, yeast expressing *CcMTP11* exhibited tolerance to Mn, Fe, and Zn, whereas *CcMTP16* conferred tolerance only to Mn and Zn (**Figures 6**, **7**). Consistent with our results, *QdMTP10.7* and *PtrMTP6* also conferred tolerance to various heavy metal stresses in yeast cells (Jiang et al., 2024; Gao et al., 2020). Notably, *CcMTP24* significantly reduced Cd accumulation in yeast (**Figure 6**), identifying it as a candidate gene for further functional analysis.

Despite the insights gained from our study, several limitations should be acknowledged. First, the Cd tolerance phenotypes observed in yeast may not fully translate to the native functions of *CcMTP* genes in planta. Future studies employing transgenic or CRISPR/Cas9-mediated approaches in *C. chinensis* would help clarify the precise mechanisms by which these MTP proteins confer heavy metal tolerance. Additionally, our functional analysis was performed specifically under Cd stress conditions; the responsiveness of *CcMTP* genes to other heavy metals, such as Mn, Fe, Cu, Co, and Zn, remains to be investigated in subsequent work.

## Conclusion

Our study provides a comprehensive identification and characterisation of the *MTP* gene family in *C. chinensis*, a species for which this gene family had remained unexplored. 25 *CcMTP* genes exhibited uneven chromosomal distribution and were classified into three major substrate-specific groups: Zn-MTPs, Zn/Fe-MTPs, and Mn-MTPs. These genes appear to have undergone gene loss events and experienced selective pressure. All CcMTPs contained conserved modified signature sequences and the cation efflux domain. Expression profiles of *CcMTP* genes across different tissues and under Cd^2+^ stress indicated their conserved and critical roles in the growth and development of *C. chinensis*. Moreover, overexpression of *CcMTP24* in the yeast mutant Δ*ycf1* enhanced Cd tolerance and reduced Cd accumulation, providing functional evidence that *CcMTP24* contributes to Cd stress resistance. In addition, *CcMTP11, CcMTP16*, and *CcMTP24* improved tolerance to multiple heavy metals, including Mn, Fe, and Zn. These findings suggest that the identified *CcMTPs* are promising molecular targets for developing Cd -tolerant *C. chinensis* cultivars, offering the dual benefit of mitigating ecological risks from heavy metal toxicity and enhancing agricultural sustainability. Further characterisation of *CcMTP*-mediated Cd transport and detoxification pathways will improve our understanding of the genetic architecture underlying metal accumulation in *C. chinensis*, thereby informing strategies for regulating metal homeostasis in medicinal plants.

## Data Availability

The original contributions presented in the study are included in the article/**Supplementary Material**. Further inquiries can be directed to the corresponding authors.

## Supplementary material

Supplementary Figure 1 Multiple sequence alignment of AtMTP and CcMTP proteins. The signature sequences and the consensus motifs HXXXD/DXXXD (where X denotes any amino acid) are highlighted by red and green open boxes, respectively.

Supplementary Figure 2 Predicted 3D models of CcMTP proteins. The structural models were computationally generated via the intensive mode of the Phyre 2 server. The resultant 3D structures were visualised using a rainbow colour scheme to depict the protein backbone from the N- to C-terminus and systematically organised according to the *CcMTP* gene family members (CcMTP1–CcMTP25).

Supplementary Figure 3 Sequence similarity network (SSN) for MTP proteins from *C. chinensis*, *A. thaliana*, and *O. sativa*. Nodes in the network represent 47 cation efflux domain-containing protein sequences from *C. chinensis*, *A. thaliana* and *O. sativa*. The SSN was constructed using EFI-EST and visualised with Cytoscape. Edges between pairs of nodes in the network correspond to two sequences ≥46% similarity.

Supplementary Figure 4 Sequence logos for 10 conserved motifs in Cation efflux domains were generated using the MEME algorithm. MEME-derived motifs are visualised as stacked amino acid letters, where the total height of each stack denotes the information content (in bits) of the corresponding site within the motif. The height of each letter within a stack reflects the product of its positional probability and the stack’s total information content. The X-axis represents motif length, whereas the Y-axis indicates the information content (in bits) for each residue.

Supplementary Figure 5 Structural distribution of conserved domains in CcMTP proteins. Cation efflux domains are denoted by green boxes, whereas zinc transporter domain and xylanase inhibitor N-terminal dimerisation domain are represented by pink and yellow boxes, respectively.
