# Supplementary figures and images for: Genomic characterisation of the metal tolerance protein gene family and elucidation of functional role in heavy metal tolerance and accumulation in Coptis chinensis
Source: Front Plant Sci. 2025 Oct 8;16:1658134. doi: 10.3389/fpls.2025.1658134 (PMC12541783; doi:10.3389/fpls.2025.1658134)

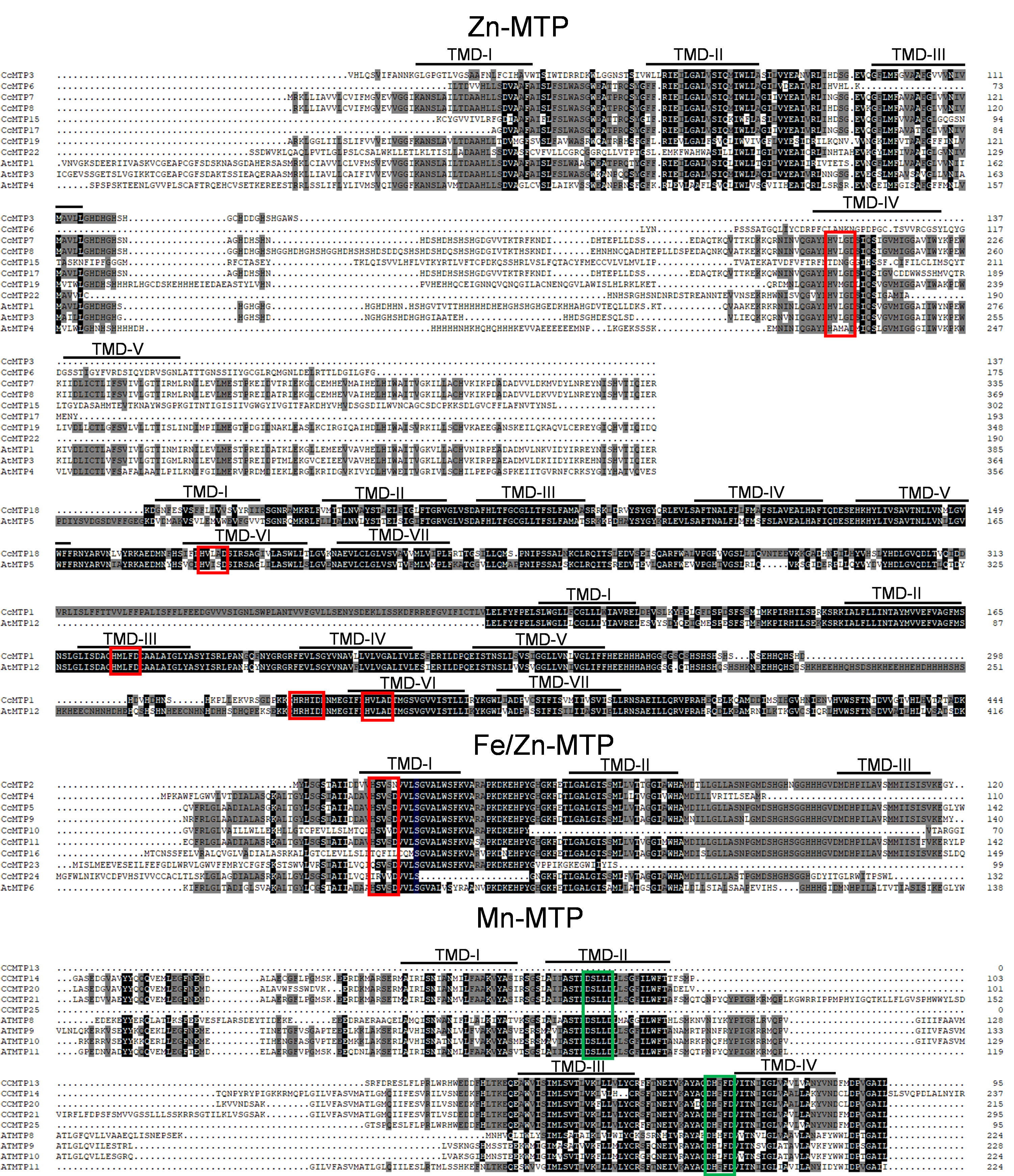

Supplement: Supplementary Figure 1 — Multiple sequence alignment of AtMTP and CcMTP proteins. The signature sequences and the consensus motifs HXXXD/DXXXD (where X denotes any amino acid) are highlighted by red and green open boxes, respectively. [file Image1.tif]

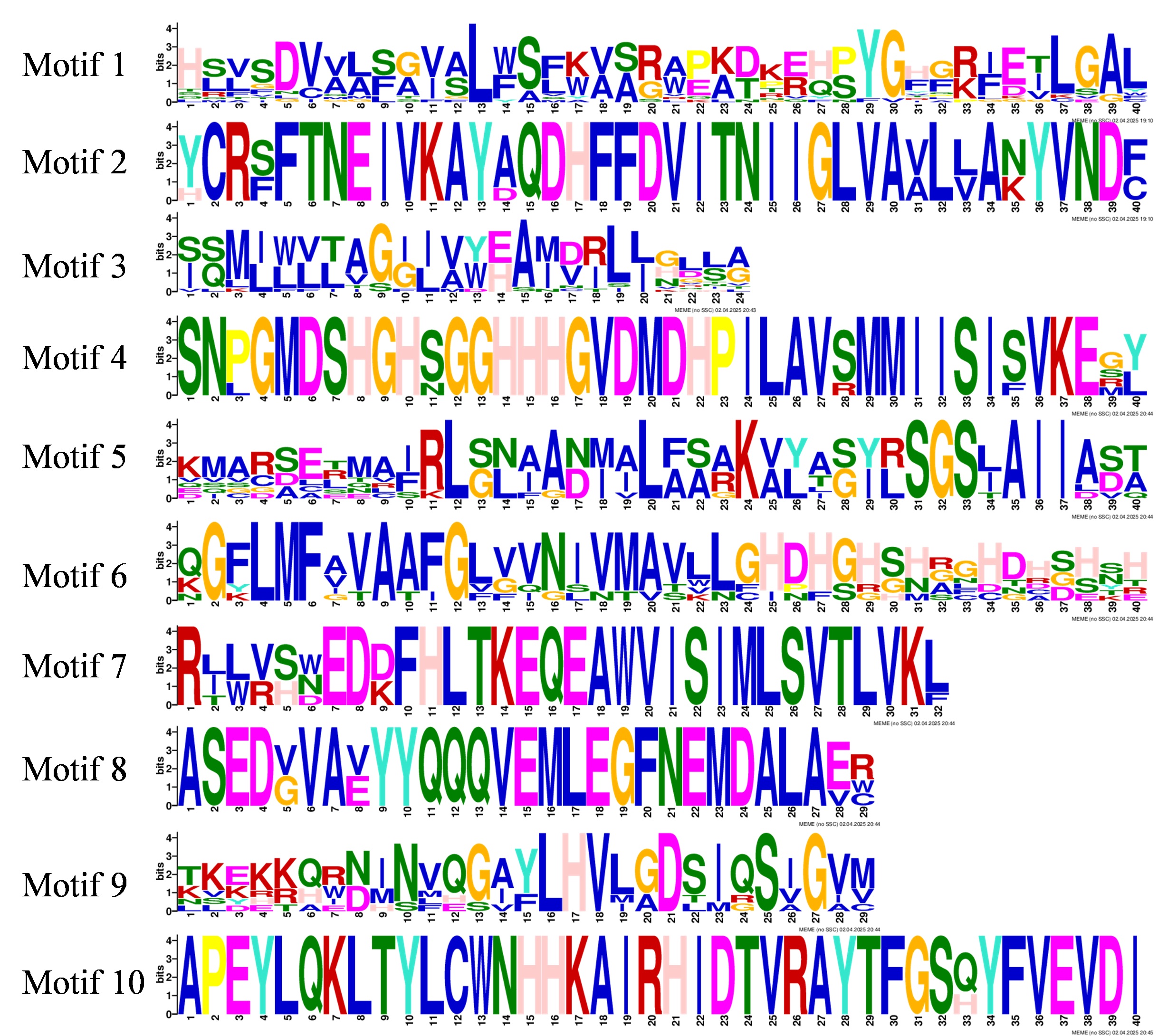

Supplement: Supplementary Figure 2 — Predicted 3D models of CcMTP proteins. The structural models were computationally generated via the intensive mode of the Phyre 2 server. The resultant 3D structures were visualised using a rainbow colour scheme to depict the protein backbone from the N- to C-terminus and systematically organised according to the CcMTP gene family members (CcMTP1–CcMTP25). [file Image2.jpeg]

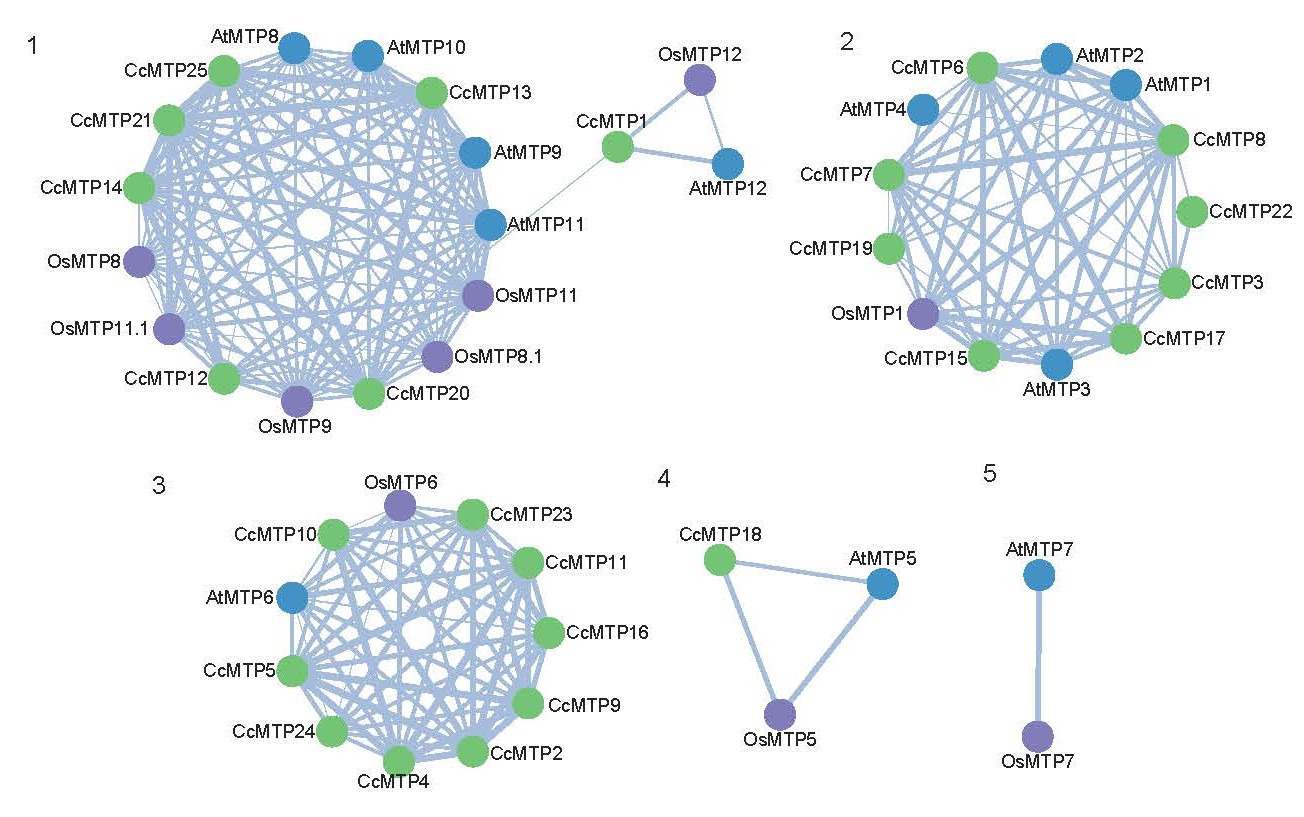

Supplement: Supplementary Figure 4 — Sequence logos for 10 conserved motifs in Cation efflux domains were generated using the MEME algorithm. MEME-derived motifs are visualised as stacked amino acid letters, where the total height of each stack denotes the information content (in bits) of the corresponding site within the motif. The height of each letter within a stack reflects the product of its positional probability and the stack’s total information content. The X-axis represents motif length, whereas the Y-axis indicates the information content (in bits) for each residue. [file Image4.jpeg]

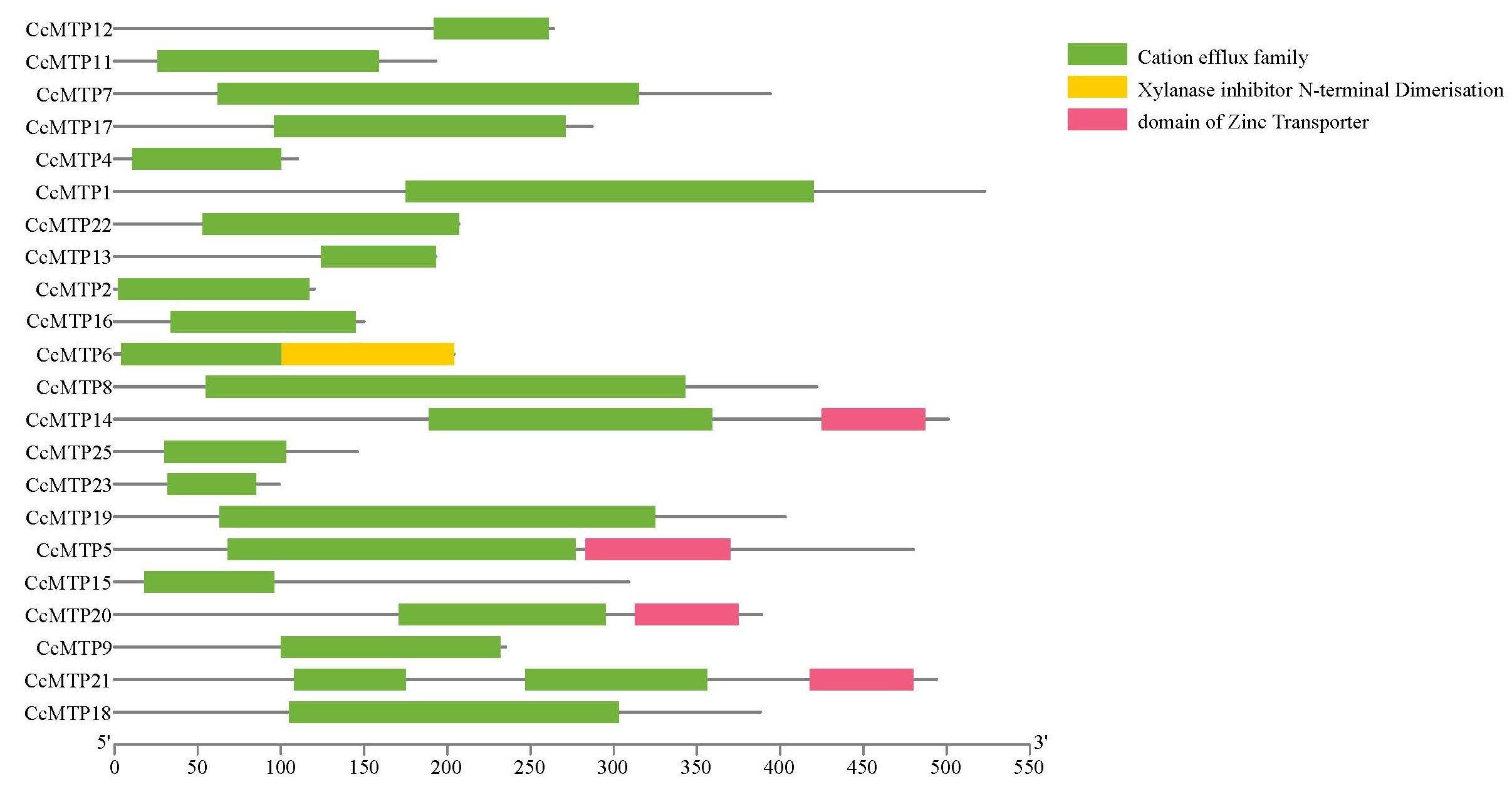

Supplement: Supplementary Figure 5 — Structural distribution of conserved domains in CcMTP proteins. Cation efflux domains are denoted by green boxes, whereas zinc transporter domain and xylanase inhibitor N-terminal dimerisation domain are represented by pink and yellow boxes, respectively. [file Image5.jpeg]
